# *Journal of NeuroEngineering and Rehabilitation* reviewer acknowledgement 2015

**DOI:** 10.1186/s12984-016-0123-z

**Published:** 2016-02-17

**Authors:** David J Reinkensmeyer

**Affiliations:** Department of Mechanical and Aerospace Engineering, University of California, Irvine, USA; Department of Anatomy and Neurobiology, University of California, Irvine, USA; Department of Biomedical Engineering, University of California, Irvine, USA; Department of Physical Medicine and Rehabilitation, University of California, Irvine, USA

## Abstract

The editors of *Journal of NeuroEngineering and Rehabilitation* would like to thank all of our reviewers who have contributed to the journal in volume 12 (2015).

**Peter Adamczyk**

United States of America

**Abidemi Bolu Ajiboye**

United States of America

**Nicholas Anderson**

United States of America

**Tomoko Aoki**

Japan

**Philippe Archambault**

Canada

**Luca Ardigo**

Italy

**Panagiotis Artemiadis**

United States of America

**Musa Audu**

United States of America

**Louis Awad**

United States of America

**Richard Baker**

United Kingdom

**Belén Ballester**

Spain

**Robert Barry**

United States of America

**Dorothy Barthélemy**

Canada

**Kilian Baur**

Switzerland

**Scott Beardsley**

United States of America

**Robin Bekrater-Bodmann**

Germany

**Juan-Manuel Belda-Lois**

Spain

**Leah Bent**

Canada

**Sergi Bermúdez I Badia**

Portugal

**Stuart Binder-Macleod**

United States of America

**Amy Blank**

United States of America

**Florian Bodranghien**

Belgium

**Marco Bonizzato**

Switzerland

**David Borton**

United States of America

**Dennis Bourbeau**

United States of America

**Jocelyn Bowden**

Australia

**Mark Bowden**

United States of America

**Elena Brown**

United States of America

**Stephen Brown**

Canada

**Cynthia Brown**

United States of America

**Szofia Bullain**

United States of America

**Rocco Calabro**

Italy

**Carmen Capo-Lugo**

United States of America

**Stephanie Carey**

United States of America

**Ilaria Carpinella**

Italy

**Amanda Carty**

Ireland

**Maura Casadio**

Italy

**Margherita Castronovo**

Germany

**Marco Cempini**

Italy

**Massimo Cenciarini**

Germany

**Andrea Cereatti**

Italy

**Benedetta Cesqui**

Italy

**Guohong Chai**

China

**Dar-Zen Chen**

Taiwan

**Maoqi Chen**

China

**Lorenzo Chiari**

Italy

**Younggeun Choi**

South Korea

**Cipriani Christian**

Italy

**Anita Christie**

United States of America

**Imre Cikajlo**

Slovenia

**Steve Collins**

United States of America

**Roberto Colombo**

Italy

**José Contreras-Vidal**

United States of America

**Louise Corben**

Australia

**Martina Coscia**

Switzerland

**Michael Crary**

United States of America

**Robert Creath**

United States of America

**Dustin Crouch**

United States of America

**William Cusack**

United States of America

**Gislins Dagnelie**

United States of America

**Frederic Danion**

France

**Koel Das**

India

**Eling D de Bruin**

Switzerland

**Carlos Alberto de Lima Braga Lopes Cordeiro**

Portugal

**Cristiano de Marchis**

Italy

**Stefano de Rossi**

United Kingdom

**Dalia de Santis**

Italy

**Jesse Dean**

United States of America

**Frederik Deconinck**

Belgium

**Antonio del Ama**

Spain

**Silvia Del-Din**

United Kingdom

**Ugo Della Croce**

Italy

**Charmaine Demanuele**

United States of America

**Nandini Deshpande**

Canada

**Ronald Deumens**

Belgium

**Volker Dietz**

Switzerland

**Dan Ding**

United States of America

**Catherine Disselhorst-Klug**

Germany

**An Do**

United States of America

**Antoinette Domingo**

United States of America

**Nick Donaldson**

United Kingdom

**Strahinja Dosen**

Germany

**Raffy Dotan**

Canada

**Edward Dougherty**

United States of America

**Marcos Duarte**

Brazil

**Sean Dukelow**

Canada

**Ayelet Dunsky**

Israel

**William Durfee**

United States of America

**Gammon Earhart**

United States of America

**Reggie Edgerton**

United States of America

**Debbie Espy**

United States of America

**Timothy Exell**

United Kingdom

**Deborah Falla**

Germany

**Susan Fasoli**

United States of America

**William Filer**

United States of America

**James Finley**

United States of America

**Lee Fisher**

United States of America

**Kevin Fite**

United States of America

**Gerard Fluet**

United States of America

**Emanuele Formento**

Switzerland

**Emily Fox**

United States of America

**Thuy Frakking**

Australia

**Antonio Frisoli**

Italy

**Armin Fuchs**

United States of America

**David Gabriel**

Canada

**Deborah Gaebler-Spira**

United States of America

**Brook Galna**

United Kingdom

**Gaetano Gargiulo**

Australia

**Roger Gassert**

Switzerland

**Marco Gazzoni**

Italy

**Sabata Gervasio**

Denmark

**Raechelle Gibson**

Canada

**Angel Gil Agudo**

Spain

**Vikash Gilja**

United States of America

**Jason Gillette**

United States of America

**Simon Giszter**

United States of America

**Joel Goebel**

United States of America

**Monica Gorassini**

Canada

**Keith Gordon**

United States of America

**Nikos Gorgoraptis**

United Kingdom

**Alena Grabowski**

United States of America

**Klaus Gramann**

Germany

**Barry Greene**

Ireland

**Josef Grohs**

Austria

**David Guiraud**

France

**Xia Guo**

Hong Kong

**Agneta Gustus**

Germany

**Nobuhiro Hagura**

Japan

**Laura Hak**

Netherlands

**Maria Hakonen**

Finland

**Andrew Hansen**

United States of America

**Levi Hargrove**

United States of America

**Christopher Hasson**

United States of America

**Malcolm Hawken**

United Kingdom

**Jacqueline Hebert**

Canada

**Tjitske Heida**

Netherlands

**Christopher Henderson**

United States of America

**John Hollman**

United States of America

**Ales Holobar**

Slovenia

**Keum-Shik Hong**

South Korea

**George Hornby**

United States of America

**Haoyuan Hsiao**

United States of America

**Xiaogang Hu**

United States of America

**Xiaoling Hu**

China

**Yuriy Hulovatyy**

United States of America

**Ianessa Humbert**

United States of America

**Christopher Hurt**

United States of America

**Leon Iasemidis**

United States of America

**Kim Ingraham**

United States of America

**Alberto Inuggi**

Italy

**Marco Iosa**

Italy

**Mark Ison**

United States of America

**Jun Izawa**

Japan

**Eric James**

United States of America

**Arno Janssen**

Netherlands

**Winnie Jensen**

Denmark

**Ning Jiang**

Germany

**Gudrun Johansson**

Sweden

**Maria Jones**

United States of America

**Deborah Kado**

United States of America

**Hiroyuki Kambara**

Japan

**Shailesh Kantak**

United States of America

**Pei-Chun Kao**

United States of America

**Kenton Kaufman**

United States of America

**Steven Kautz**

United States of America

**Robert Kearney**

Canada

**Thierry Keller**

Spain

**Spencer Kellis**

United States of America

**Stephen Kelly**

United Kingdom

**Laurence Kenney**

United Kingdom

**Georg Kerkhoff**

Germany

**Sohee Kim**

South Korea

**Christine King**

United States of America

**Lee Kirby**

Canada

**Rachel Kizony**

Israel

**Verena Klamroth-Marganska**

Switzerland

**Cliff Klein**

Canada

**Julius Klein**

Spain

**Rudolf Klemetti**

Finland

**Jonathan Kofman**

Canada

**Yasuharu Koike**

Japan

**Juergen Konczak**

United States of America

**Mervi Könönen**

Finland

**Srinivas Kota**

United States of America

**Dean Krusienski**

United States of America

**Masayoshi Kubo**

Japan

**Jason Kutch**

United States of America

**Vincenzo La Bella**

Italy

**Maria Marcella Lagana**

Italy

**Olivier Lambercy**

Switzerland

**Linnea Larsson**

Sweden

**Kate Laver**

Australia

**Seung-Han Lee**

South Korea

**Sunghoon Ivan Lee**

United States of America

**Yun-Ju Lee**

United States of America

**Hyunglae Lee**

United States of America

**Beom Chan Lee**

United States of America

**Sang Wook Lee**

United States of America

**Olive Lennon**

Ireland

**Mindy Levin**

Canada

**Guanglin Li**

China

**Sheng Li**

United States of America

**Jing-Nong Liang**

United States of America

**Dario G Liebermann**

Israel

**Sook-Lei Liew**

United States of America

**Chou-Ching Lin**

Taiwan

**Jack Lin**

United States of America

**Jie Liu**

United States of America

**Roberto Lloréns**

Spain

**Jason Long**

United States of America

**Luca Lonini**

United States of America

**Eduardo López-Larraz**

Spain

**Beth Lopour**

United States of America

**Madeleine Lowery**

Ireland

**Peter Lum**

United States of America

**Inbal Maidan**

Israel

**Nathan Makowski**

United States of America

**Philippe Malcolm**

Belgium

**Nebojsa Malesevic**

Serbia

**Khondaker A Mamun**

Bangladesh

**Andrea Mannini**

Italy

**Paul Marasco**

United States of America

**Laura Marchal-Crespo**

Switzerland

**Paul Marshall**

Australia

**Clarisa Martinez**

United States of America

**Kei Masani**

Canada

**Claudia Mazzà**

Italy

**Pietro Mazzoni**

United States of America

**Sanford Meek**

United States of America

**Sabato Mellone**

Italy

**Rochelle Mendonca**

United States of America

**Alma Merians**

United States of America

**Peter Meulenbroek**

United States of America

**Marie-Helene Milot**

Canada

**Marco Alessandro Minetto**

Italy

**Anat Mirelman**

Israel

**Koji Miyashita**

Japan

**Joseph Mizrahi**

Israel

**Filippo Molinari**

Italy

**Kamyar Momeni**

United States of America

**Juan C Moreno**

Spain

**David M Morris**

United States of America

**Denis Mottet**

France

**Silvia Muceli**

Germany

**Mukul Mukherjee**

United States of America

**Bernadette Murphy**

Canada

**Kristin Musselman**

Canada

**Aniket Nagle**

Switzerland

**Antonio Nardone**

Italy

**Nathan Neckel**

United States of America

**Zoran Nenadic**

United States of America

**Alice Nieuwboer**

Belgium

**Shinya Ogaya**

Japan

**Taher Omari**

Australia

**Kristine Oostra**

Belgium

**Max Ortiz-Catalan**

Sweden

**Rieko Osu**

Japan

**Min-Chun Pan**

Taiwan

**Enrica Papi**

United Kingdom

**Ilaria Pasciuto**

Italy

**Carolynn Patten**

United States of America

**Matthew Patterson**

Ireland

**Lucie Pelland**

Canada

**Hugo Pereira**

United States of America

**Elisabetta Peri**

Italy

**Angkoon Phinyomark**

Canada

**Rakesh Pilkar**

United States of America

**José Eduardo Pompeu**

Brazil

**Jim Potvin**

Canada

**Girijesh Prasad**

United Kingdom

**Erik Prinsen**

Netherlands

**Tariq Rahman**

United States of America

**Georg Rauter**

Switzerland

**Rafael Raya**

Spain

**Stephen Redmond**

Australia

**Daniel Renjewski**

Switzerland

**Philip Requejo**

United States of America

**Iman Rezazadeh**

United States of America

**Christopher Rhea**

United States of America

**Aidan Roche**

Austria

**Eduardo Rocon**

Spain

**Nancy Rodriguez**

France

**Eric Rombokas**

United States of America

**Renaud Ronsse**

Belgium

**Margaret Roos**

United States of America

**Justin Rowe**

United States of America

**Jennifer Ryan**

United Kingdom

**Arash Salarain**

Switzerland

**Jose Salazar-Torres**

United Kingdom

**Brian Sandroff**

United States of America

**Vittorio Sanguineti**

Italy

**Marco Santello**

United States of America

**Lumy Sawaki**

United States of America

**Gregory Sawicki**

United States of America

**Edward Sazonov**

United States of America

**Sydney Schaefer**

United States of America

**Thomas Schauer**

Germany

**Erik Scheme**

Canada

**Maurizio Schmid**

Italy

**Brian Schmit**

United States of America

**Erich Schneider**

Germany

**Stephen Scott**

Canada

**Ervin Sejdic**

United States of America

**Masaki Sekine**

Japan

**Jon Sensinger**

Canada

**Nah Seo**

United States of America

**Lauren Sergio**

Canada

**Pinata Sessoms**

United States of America

**Giacomo Severini**

Ireland

**Nitin Sharma**

United States of America

**Kelli Sharp**

United States of America

**Susan Shaw**

United States of America

**Safa Shehab**

United Arab Emirates

**Roberta Shepherd**

Australia

**Kathryn Sibley**

Canada

**C Douglas Simmons**

United States of America

**Ann Simon**

United States of America

**Martin Simoneau**

Canada

**Tarkeshwar Singh**

United States of America

**Sue Ann Sisto**

United States of America

**Brooke Slavens**

United States of America

**Marc Slutzky**

United States of America

**Surjo R Soekadar**

Germany

**Jacob Sosnoff**

United States of America

**William Speier**

United States of America

**Ann Spungen**

United States of America

**Shraddha Srivastava**

United States of America

**Penny Standen**

United Kingdom

**Katherine Steele**

United States of America

**Dick F Stegeman**

Netherlands

**Jill Stewart**

United States of America

**Arno Stienen**

Netherlands

**Leia Stirling**

United States of America

**Ian Stokes**

United States of America

**Oliver Stoller**

Switzerland

**Mary Stoykov**

United States of America

**Sofia Straudi**

Italy

**Vignesh Subbian**

United States of America

**Susanna Summa**

Italy

**Eva Swinnen**

Belgium

**Stephan Swinnen**

Belgium

**Kota Takahashi**

United States of America

**Dawn Taylor**

United States of America

**Scott Telfer**

United Kingdom

**Claudius Thome**

Austria

**Rune Thorsen**

Italy

**Annick Timmermans**

Belgium

**Julia Totosy de Zepetnek**

Canada

**Diana Trojaniello**

Italy

**Catherine Trombly Latham**

United States of America

**Andrea Turolla**

Italy

**Michel Vacher**

France

**Nikola Valchev**

Netherlands

**Rita van den Berg-Emons**

Netherlands

**Loek van der Heide**

Netherlands

**Jaap van Dieen**

Netherlands

**Joost van Kordelaar**

Netherlands

**Vassilios Vardaxis**

United States of America

**Peter Veltink**

Netherlands

**Gloria Vergara Diaz**

United States of America

**Johanna Wagner**

Austria

**Tim Wagner**

United States of America

**Conor J Walsh**

United States of America

**Chunji Wang**

United States of America

**Po Wang**

Germany

**Takashi Watanabe**

Japan

**Aner Weiss**

Israel

**Jianhua Wu**

United States of America

**Ming Wu**

United States of America

**Hong-Bo Xie**

Australia

**Melody Ying-Yu Huang**

Switzerland

**Tejin Yoon**

United States of America

**Scott Youmans**

United States of America

**Aaron Young**

United States of America

**E Paul Zehr**

Canada

**Karl Zelik**

United States of America

**Erika Zemková**

Slovakia

**Xu Zhang**

China

**Mingming Zhang**

New Zealand

**Ting Zhang**

United States of America

**Fan Zhang**

United States of America

**Xiaorong Zhang**

United States of America

**Wangming Zhang**

China

**Xiaorong Zhang**

United States of America

**Xu Zhang**

China

**Kristin Zhao**

United States of America

**Guohun Zhu**

China

